# Substance P Administered after Myocardial Infarction Upregulates Microphthalmia-Associated Transcription Factor, GATA4, and the Expansion of c-Kit^+^ Cells

**DOI:** 10.1155/2020/1835950

**Published:** 2020-02-10

**Authors:** Yun-Mi Jeong, Xian Wu Cheng, Weon Kim

**Affiliations:** ^1^Division of Cardiology, Department of Internal Medicine, Kyung Hee University Hospital, Kyung Hee University, Seoul, Republic of Korea; ^2^Department of Mechanical Engineering, Korea Polytechnic University, 237 Sangidaehak Street, Si-heung City, Republic of Korea; ^3^The Department of Cardiology, Yanbian University Hospital, Yanji, China

## Abstract

Microphthalmia-associated transcription factor (MITF), a basic helix-loop-helix leucine zipper transcription factor, can govern gene expression by binding to E box elements in the promoter region of its target gene. Although high levels of MITF have been observed in cardiomyocytes and the heart, the role of MITF after myocardial infarction (MI) remains unclear. We investigated the association between substance P (SP)/neurokinin-1 receptor (NK_1_R) signaling and MITF expression after MI. Male Sprague-Dawley rats (8 weeks) were randomly divided in two groups: ischemia/reperfusion injury (I/R) and SP injection (5 nmol/kg, SP+I/R). At the end of 7 days, the left ventricle (LV; LV^7daysI/R^, LV^7daysSP+I/R^) and infarct-related areas (IA; IA^7daysI/R^, IA^7daysSP+I/R^) from the hearts were collected. Immunofluorescence staining demonstrated that the LV^7daysSP+I/R^ had a larger population of c-Kit^+^ GATA4^high^ cells, which markedly upregulated MITF, c-Kit, and GATA4. c-Kit^+^ cells in the explant-derived cells (EDCs) derived from IA^7daysSP+I/R^ migrated more widely than EDCs IA^7daysI/R^. Immunofluorescence staining, western blot analysis, and qRT-PCR assay showed that SP-treated c-Kit^+^ cells exhibited a high expression of c-Kit, GATA4, and MITF. FTY720 (a MITF inhibitor), RP67580 (NK_1_R inhibitor), or both inhibited the migration and proliferation of c-Kit^+^ cells increased by SP and blocked the upregulation of c-Kit, GATA4, and MITF. Overall, we suggest that MITF might be a potential regulator in SP-mediated c-Kit^+^ cell expansion post-MI via c-Kit and GATA4.

## 1. Introduction

Heart failure (HF) is expected to grow as an important clinical and public health challenge. HF occurs in response to hypertension and myocardial infarction from coronary artery disease, consequently giving rise to left ventricular (LV) remodeling [1, 2]. Although the mechanisms underlying cardiac growth, cardiac hypertrophy, and LV remodeling are still poorly understood, their intricate interactions might involve endogenous resident c-Kit^+^ cells and cardiomyocytes lost through direct injury [3–5]. For example, endothelin-1, angiotensin II, natriuretic peptides, and/or *α*1 and *β*-adrenergic agonists might play a substantial modulatory role in cardiac remodeling [4]. Cardiac transcription factors (such as GATA4, Nkx2.5, TBX5, and MEF2) and chromatin remodeling enzymes are also involved in stress regulation of the adult heart [5, 6]. Therefore, gaining a more precise understanding of the molecular mechanisms governing transcriptional regulation during postinfarct LV remodeling would be helpful in the development of novel strategies for HF therapies.

Microphthalmia-associated transcription factor (MITF), a basic helix-loop-helix leucine zipper DNA-binding protein, is known as a master transcription factor that has been detected in various cell types, such as melanocytes, mast cells, and osteoclasts [7–10]. In case of the heart, MITF-H, the heart-specific isoform, is abundantly expressed in cardiomyocytes and is important in the hypertrophic response [7–10]. MITF-mutated mice (MITF^ce/ce^ mice with the ce/ce mutation lack the Zip domain of MITF) show dramatically reduced heart weight/body weight ratios, systolic function, and cardiac output compared to wild-type mice [7]. A previous study has demonstrated that MITF regulates cardiac hypertrophy through targeting miR-541 [10]. According to these studies, MITF is critical for the cardiac growth and hypertrophic response, though only a few genes and signaling pathways associated with MITF have been recently identified [7–10].

Neuropeptide substance P (SP) is an undecapeptide member of the tachykinin neuropeptide family [11–14]. Studies have found SP to function as a neuromodulator for many biological processes through neurokinin-1 receptor (NK_1_R) [11–14]. Previous research describes the important roles of SP/NK_1_R in the cardiovascular system [11–14]. Several studies note the cardioprotective effects of SP on ischemia/reperfusion injury (I/R) [11–14]. However, the role of SP/NK_1_R signaling during cardiac repair after I/R remains unclear. Our previous study demonstrates that SP prevents I/R by modulating stem cell mobilization [12]. The present study investigates how exogenous SP affects the healing of a damaged LV after I/R. To accomplish this, we analyzed cardiac stem cell-specific gene expression and related factors in the LV and infarct-related areas (IA) of I/R rats with or without SP treatment.

## 2. Materials and Methods

### 2.1. Materials

SP (6.7 *μ*g/kg/0.1 ml) and FTY720 (a sphingosine 1-phosphate receptor agonist that inhibits the expression of MITF) [15] were purchased from Sigma (St. Louis, MO, USA). RP67580 (RP, a selective nonpeptide tachykinin NK_1_R antagonist, 1 mg/kg) was obtained from R&D Systems Inc. (Minneapolis, MN, USA). Phospho-MITF (ser180) antibody, Hoechst 33342, and DAPI were purchased from Thermo Fisher Scientific (Rockford, IL, USA). AccuPower® RocketScript™ Cycle RT PreMix (dN12) and AccuPower® ProFi Taq PCR PreMix were purchased from Bioneer (Daejeon, Korea). SYBR® Green Mix was obtained from Applied Biosystems (Lincoln, CA, USA). Antibodies specific for c-Kit (sc5535), NK_1_R (sc15323), MITF (sc56725), and actin (sc47778) were obtained from Santa Cruz Biotechnology, Inc. (Santa Cruz, CA, USA). Antibodies that recognize GATA4 (ab86371) and Alexa Fluor® secondary antibodies were purchased from Abcam (Cambridge, UK). Another antibody specific for c-Kit (phosphor Tyr568/Tyr570) was obtained from GeneTex (Isleworth, UK). Phospho-Akt (4060S) and Akt were purchased from Cell Signaling Technology (Beverly, USA).

### 2.2. Animal Models and *Ex Vivo* Explant Outgrowth Culture Assay

All the experiments on the eight-week-old male Sprague-Dawley (SD) rats were carried out according to the Institutional Animal Care and Ethics Committee of Kyung Hee Medical Center (KHMC-IACUC:2015-028). The SD rats were randomly divided into 2 groups (*n* = 22 each): I/R and I/R with 5 nmol/kg SP injection (SP+I/R). The left anterior descending coronary artery was occluded for 40 min followed by 7 days reperfusion with and without SP. The rats were euthanized, and the LV (LV^7daysI/R^, LV^7daysSP+I/R^) derived from heart samples were collected. IA tissue was cut into 1 to 2 mm fragments, washed with Ca^2+^/Mg^2+^-free PBS, and digested three times for 10 min with 0.2% trypsin and 0.1% collagenase at 37°C. IA fragments were incubated with complete medium (CM; Dulbecco's modified Eagle's medium supplemented with 10% ES cell grade FBS, 5% horse serum, 10 ng/ml LIF, 1% penicillin-streptomycin, fungizone, and gentamicin) at 37°C in a 5% CO_2_ incubator. After 2 weeks, the attached cells, which migrated out and surrounded the explants, were analyzed by immunofluorescence staining (IFS) with c-Kit antibodies. The c-Kit^+^ cells were purified by a magnet-activated cell sorting (MACS) system (Dynal Biotech, Oslo, Norway). Explant-derived cells (EDCs) were suspended in trypsin, incubated with a rabbit anti-c-Kit antibody (1 : 100), and separated using immunomagnetic microbeads (Dynal Biotech). c-Kit^+^ cells were cultured for 1 month with CM at 37°C in a 5% CO_2_ incubator.

### 2.3. c-Kit^+^ Cell Proliferation Assay

The c-Kit^+^ cell proliferation assay was assessed using an EZ-Cytox cell viability assay kit (DoGEN, Seoul, Korea) [16]. The c-Kit^+^ cells were pretreated with FTY720 (5 *μ*M) or RP67580 (20 *μ*g/ml) or both for 2 h. After SP treatment for 7 days, the cultured medium was removed. Cells were stained with EZ-Cytox solution for 1 h. Absorbance was determined at 490 nm using an ELISA reader (EMax; Molecular Devices, Sunnyvale, CA, USA).

### 2.4. c-Kit^+^ Cell Migration Assay

The cell migration assay was performed using 0.8 *μ*m pore size and 24-well transwell migration chambers coated with type IV collagen (10 *μ*g/ml) as previously described [16]. 1 × 10^4^ c-Kit^+^ cells were seeded into the upper transwell chambers containing medium without FTY720 or RP67580 or both. Then, the chamber was inserted into each well of 24-well plates containing 600 *μ*l medium supplemented with or without SP at the indicated concentration in the presence or absence of FTY720 (5 *μ*M) or RP67580 (20 *μ*g/ml) or both. The chambers were then incubated for 24 h at 37°C in a 5% CO_2_ incubator. The cells that migrated through to the outer side of the membrane were stained with a crystal violet staining solution. The absorbance was determined at 590 nm using an ELISA reader (EMax; Molecular Devices, Sunnyvale, CA, USA).

### 2.5. Quantitative Reverse-Transcription PCR (qRT-PCR)

cDNA was synthesized using AccuPower® RocketScript™ Cycle RT PreMix (dN12) (Bioneer, Daejeon, Korea). qRT-PCR assays were carried out with SYBR® Green Mix and the appropriate primers (Applied Biosystems) and were run on a StepOnePlus real-time PCR system (Applied Biosystems). The relative gene expression from all data was obtained using the *Δ*Ct method with normalization versus RPL-32 as previously described [16]. The primers used were as follows: c-Kit (forward: AGACGTACAGATCCAGAATG, reverse: TGCTCTTTGCTGTTACCTT); GATA4 (forward: ACCCTGCGAGACACCCCAAT, reverse: GTAGAGGCCACAGGCGTTGC); MITF (forward: CATCACGCATCTTGCTACGC, reverse: TGCATGAACTGGGCTGCCTG); and RPL-32 (forward: TGTCAAGGAGCTGGAAGTGC, reverse: AGGCACACAAGCCATCTATTCA).

### 2.6. IFS and Confocal Microscopy

The LVs were fixed with 4% paraformaldehyde (PFA) and paraffin embedded, sectioned into 7 *μ*m thick sections, and stained with standard IFS methods as previously described [10, 17]. To detect the expression of c-Kit or MITF after SP treatment, c-Kit^+^ cells were treated with SP (10 nM). After SP treatment for 1 day, the cultured medium was removed. After fixing in 4% PFA at 4°C for 15 min, cells were washed with PBS, permeabilized with 5% BSA and 0.1% Triton X-100, and incubated with primary antibodies. After nuclear DAPI and Hoechst staining, immunostained cells were imaged using an inverted Zeiss Axio Observer Z1 confocal microscope with 405, 458, 488, 514, 561, and 633 nm laser lines. All images were selected with sample identities blinded, and at least 20 random images were obtained from each well or group.

### 2.7. Western Blot Analysis

The frozen samples were disrupted using the TissueLyser II (Qiagen), after which an ice-cold PRP-PREP protein extraction solution with a protease inhibitor cocktail (iNtRON Biotechnology, Inc., Seoul, Korea) was added, and the samples were homogenized with stainless steel beads (Qiagen, CA, USA). Protein concentration was assessed using a BCA kit (Thermo Scientific, Rockford, IL, USA). An equal amount of protein (80 *μ*g) from each sample was loaded onto 10% to 12% SDS gel and transferred to a PVDF membrane (Merck Millipore, MA, USA). The membranes were blocked for 2 h at room temperature with 5% nonfat dry milk in PBS containing 0.1% Tween-20 and incubated with primary antibodies (1 : 1000 and 1 : 500, respectively) overnight at 4°C. After washing three times, the membranes were incubated with a horseradish peroxidase-conjugated secondary antibody (1 : 5000) at RT for 2 h and visualized with a chemiluminescence substrate.

### 2.8. Statistical Analysis

Student's *t-*tests (for comparisons between two groups) or a one-way analysis of variance (ANOVA) (for comparisons among three or more groups) followed by Tukey post hoc tests was used for the statistical analyses. SPSS software ver. 17.0 (SPSS, Chicago, IL) was used. A value of *P* < 0.05 was considered significant. Data are expressed as means ± standard error (SE). ^∗^*P* < 0.05–0.01, ^∗∗^*P* < 0.01–0.001, and ^∗∗∗^*P* < 0.001 vs. corresponding controls. All error bars represent the standard deviation of three or more biological replicates.

## 3. Results

### 3.1. SP Increases the Endogenous Resident c-Kit^+^ GATA^high^ Cells and Upregulates c-Kit, GATA4, and MITF in I/R-Injured LV

**Figure 1:**
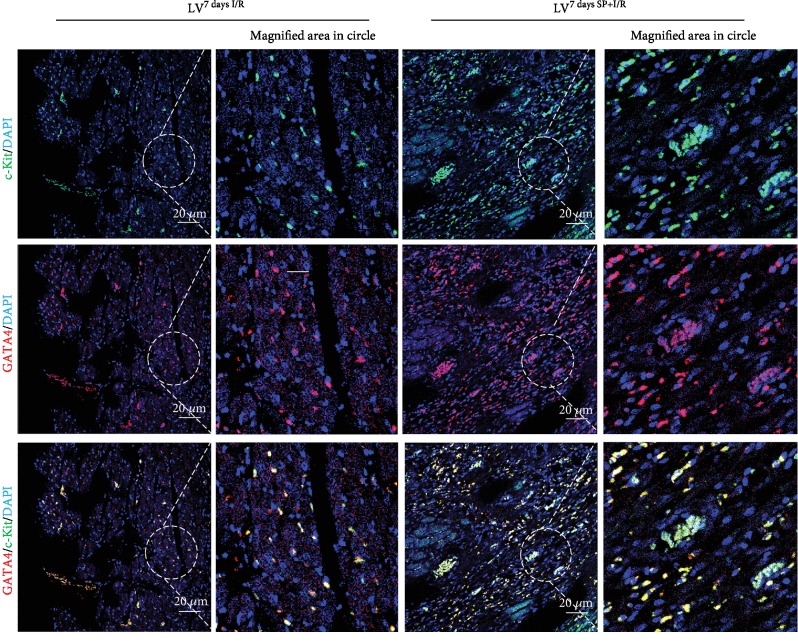
LV^7daysSP+I/R^ shows c-Kit^+^ GATA^high^ cell enrichment. Confocal images of GATA4 staining (red fluorescence) in conjunction with c-Kit (green fluorescence) and nuclear staining (blue fluorescence) in tissue sections of LV^7daysI/R^ and LV^7daysSP+I/R^. Scale bars, 20 *μ*m with a further magnified area indicated by the dotted white circle.

**Figure 2:**
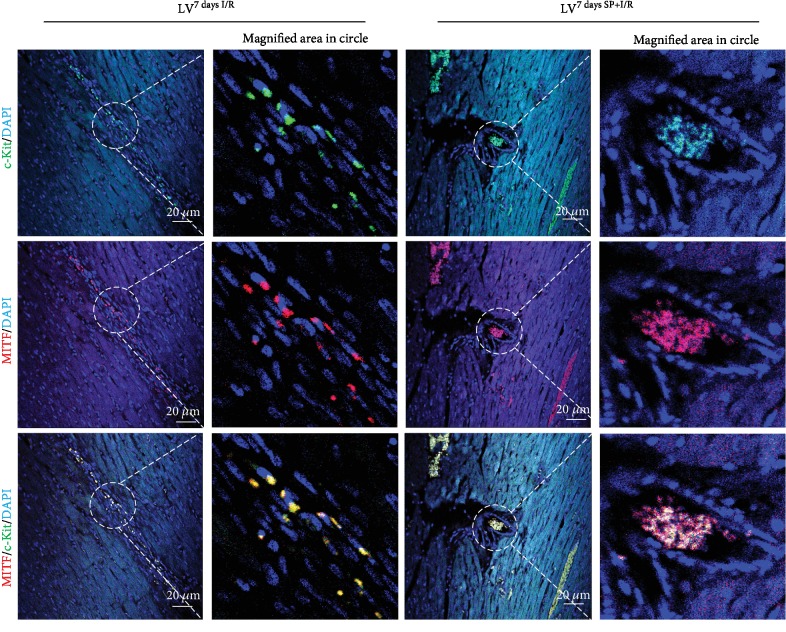
A high coexpression of MITF and c-Kit in LV^7daysI/R^ and c-LV^7daysSP+I/R^. Confocal images of MITF staining (red fluorescence) in conjunction with c-Kit (green fluorescence) and nuclear staining (blue fluorescence) in sections of LV^7daysI/R^ and LV^7daysSP+I/R^. Scale bars, 20 *μ*m with a further magnified area indicated by the dotted white circle.

**Figure 3:**
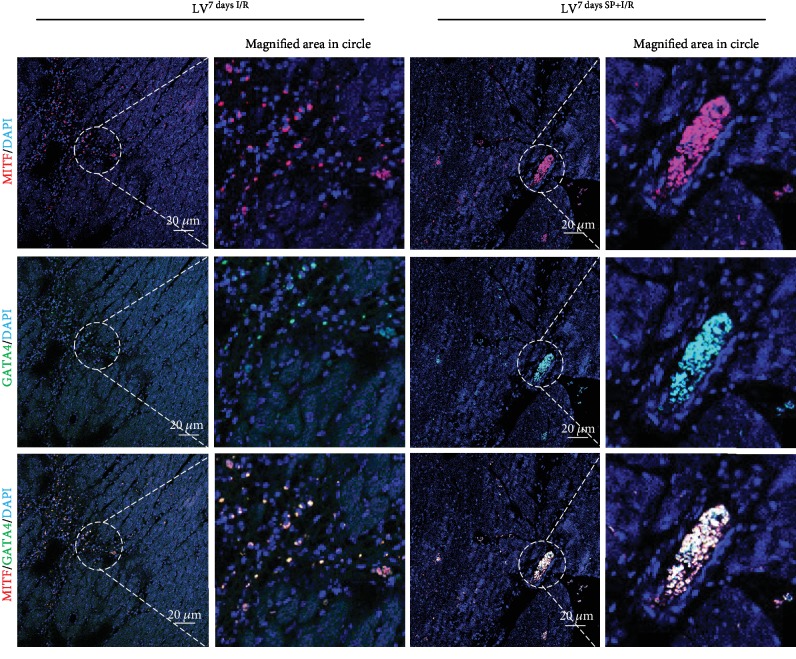
Confocal analysis of MITF and GATA4 in LV^7daysI/R^ and c-LV^7daysSP+I/R^. Confocal images of MITF staining (red fluorescence) in conjunction with GATA4 (green fluorescence) and nuclear staining (blue fluorescence) in sections of LV^7daysI/R^ and LV^7daysSP+I/R^. Scale bars, 20 *μ*m with a further magnified area indicated by the dotted white circle.

**Figure 4:**
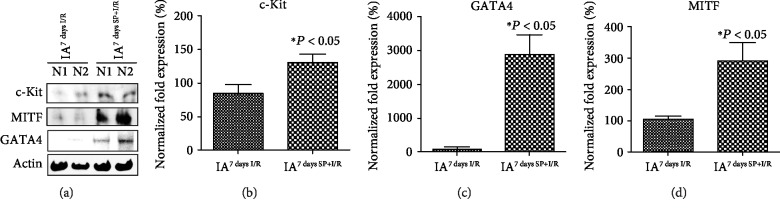
High expression of MITF, c-Kit, and GATA4 in IA^7daysSP+I/R^. (a) Western blot analysis results for the IA^7daysI/R^ and IA^7daysSP+I/R^ rats. (b–d) qRT-PCR analysis of relative mRNA levels of the targeted genes and ^∗^*P* < 0.05 versus corresponding controls using Student's *t*-test.

The mechanism by which SP contributes to the expansion of c-Kit^+^ cells after MI [14] was analyzed LV^7daysI/R^ and LV^7daysSP+I/R^ using IFS with c-Kit and GATA4 antibodies. Confocal images showed an increase of c-Kit^+^ GATA4^+^-expressed cells in LV^7daysSP+I/R^ compared to LV^7daysI/R^ (Figure 1). The expression of c-Kit and GATA4 is important for promoting growth and migration of c-Kit^+^ cells [17]. In addition, several studies of I/R-injured hearts have suggested that the number of c-Kit^+^ cells increases due to pressure overload-induced cardiac hypertrophy [17]. Representative confocal images show the coexpression of MITF and GATA4 or c-Kit in LV^7daysI/R^ and LV^7daysSP+I/R^, indicating a high level of MITF expression by SP treatment (Figures 2 and 3). MITF plays a key role in the pathway leading to cardiac growth and the hypertrophic response in the heart (6–10). However, very little is known about the molecular mechanism of MITF after MI. Next, we hypothesized that if SP/NK_1_R signaling leads to the activation of endogenous resident c-Kit^+^ GATA4^+^ cells, the expression of c-Kit, GATA4, and MITF might be involved. To examine this, we separated IA^7daysI/R^ and IA^7daysSP+I/R^ from LV^7daysI/R^ and LV^7daysSP+I/R^. The expression levels of c-Kit, MITF, and GATA4 at the mRNA and protein levels were measured by qRT-PCR and western blot analysis. These analyses revealed a significant increase in c-Kit, GATA4, and MITF expression in LV^7daysSP+I/R^ compared to LV^7daysI/R^ (Figure 4). In particular, GATA expression in LV^7daysSP+I/R^ at the mRNA level markedly increased (Figure 4(c)). These results provide evidence that the molecular pathways of SP might induce the expansion of endogenous resident c-Kit^+^ GATA4^high^-expressed cells, which are associated with c-Kit, GATA4, and MITF expression.

### 3.2. MITF Plays an Important Role in SP-Promoted Proliferation and Migration of c-Kit^+^ GATA^high^ Cells

**Figure 5:**
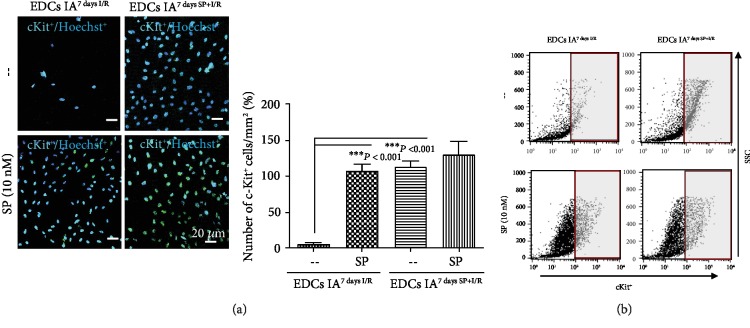
Expansion of c-Kit^+^ cells in IA^7daysI/R^ and IA^7daysSP+I/R^ with or without SP. (a) Confocal fluorescence images showing c-Kit-expressing (green) cells in each group. Hoechst 33342 (blue) allowed visualization of the nucleus in cells. Scale bars, 20 *μ*m. Graphs of the number of c-Kit^+^ cells/mm^2^ (%) in each group expressed as a percentage difference compared to the corresponding control. ^∗∗∗^*P* < 0.001 versus corresponding control using Student's *t*-test. (b) Representative dot plot of FACS analysis of EDCs derived from IA^7daysI/R^ and IA^7daysSP+I/R^ in the presence or absence of SP (10 nM) using c-Kit antibodies. Negative controls indicated as the assessment of nonspecific binding of Alexa Fluor®488 antibodies to c-Kit^+^ cells.

**Figure 6:**
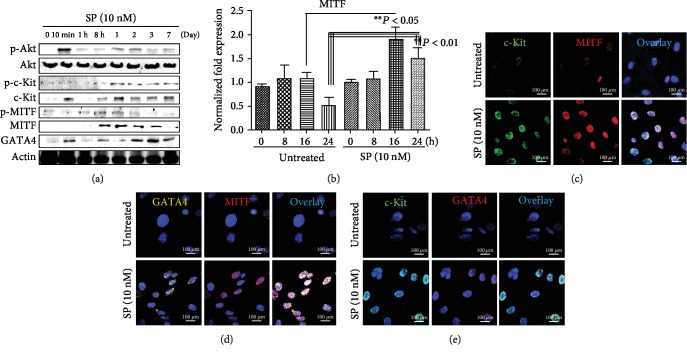
SP induces the expression of MITF in c-Kit^+^ cells. (a) Western blot analysis showing the expression of targeted proteins in SP-treated c-Kit^+^ cells over time. (b) qRT-PCR analysis of relative mRNA levels of MITF in c-Kit^+^ cells after SP treatment (10 nM) at the indicated time points. ^∗^*P* < 0.05 or ^∗∗^*P* < 0.01 versus corresponding controls using Student's *t*-test. (c–e) Confocal microscopy images showing c-Kit, MITF, and GATA4 and dual staining of these targeted proteins in untreated and SP-treated c-Kit^+^ cells. DAPI (blue) allowed visualization of the nucleus in cells. Scale bars, 100 *μ*m.

**Figure 7:**
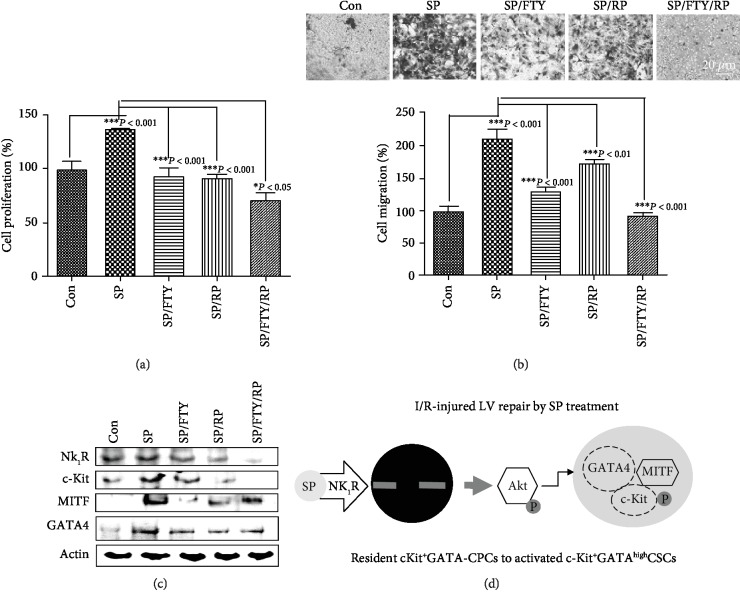
The effects of FTY720 and RP67580 on SP-treated c-Kit^+^ cells. (a) Graph indicating the proliferation of c-Kit^+^ cells with one or both of these inhibitors, as identified using the EZ-Cytox cell viability assay kit. (b) Phase-contrast images of transwell migration assay for control and SP treatment with one or both of these inhibitors. Graph indicating the rate of cell migration. ^∗^*P* < 0.05, ^∗∗^*P* < 0.01, or ^∗∗^*P* < 0.001 versus corresponding controls using one-way analysis of variance (ANOVA) followed by Tukey's post hoc tests or Student's *t*-test. (c) The effect of FTY720 and RP67580 on SP-stimulated c-Kit^+^ cells pretreated with one or both of these inhibitors. Actin is the loading control. (d) Proposed model for the molecular mechanism of SP in LV repair post I/R.

If SP/NK_1_R signaling positively regulates the endogenous resident c-Kit^+^ cells in IA through c-Kit, GATA4, and MITF, then many more c-Kit^+^ cells from IA^7daysSP+I/R^ may migrate than from IA^7daysI/R^. To test this, IA^7daysI/R^ and IA^7daysSP+I/R^ fragments were cultured using an *ex vivo* explant outgrowth assay. Expanded c-Kit^+^ cells were characterized by confocal microscopy. Confocal images and FACS analysis demonstrated that migrated c-Kit^+^ cells from IA^7daysSP+I/R^ EDCs were significantly higher than from IA^7daysI/R^ in the absence and presence of additional SP treatment (Figure 5). To determine whether SP affects the expression of MITF and GATA4 in c-Kit^+^ cells, we purified c-Kit^+^ cells from IA^7daysI/R^ EDCs using MACS methods with c-Kit antibodies. Purified c-Kit^+^ cells were treated with SP at the indicated time points. As shown in Figure 6(a), SP-treated c-Kit^+^ cells markedly activated the phosphorylation of Akt at 10 min and enhanced the phosphorylation of c-Kit and MITF at 8 h. The expression of c-Kit, GATA4, and MITF induced by SP subsequently increased at 8 h (Figure 6(a)). qRT-PCR showed an elevated expression of MITF at 16 h (Figure 6(b)). Confocal images further confirmed that the location and expression of MITF considerably overlapped with c-Kit or GATA4 expression (Figures 6(c)–6(e)). We next used RP67589 and FTY720 to further verify whether the expansion of c-Kit^+^ cells was at least in part due to SP/NK_1_R signaling and MITF expression. These inhibitors both alone and in combination significantly inhibited the enhancement of cell proliferation and migration induced by SP (Figures 7(a) and 7(b)). In addition, these inhibitors alone and together prevented the upregulation of c-Kit, GATA4, and MITF in SP-treated c-Kit^+^ cells at mRNA and protein levels (Figure 7(c), Fig. [Supplementary-material supplementary-material-1] and Fig. [Supplementary-material supplementary-material-1]). Therefore, these observations indicate that SP/NK_1_R signaling-accelerated c-Kit^+^ cell expansion might relate to c-Kit, GATA4, and MITF expression.

## 4. Discussion

In the present study, we have identified an abundance of c-Kit^+^ GATA4^high^ cells in IA^7daysI/R^ after SP treatment. The expression of MITF and GATA4 at the protein and mRNA levels in IA^7daysSP+I/R^ and SP-treated c-Kit^+^ cells was dramatically increased. Although the specific roles of MITF and GATA4 in cardiac repair have not yet been validated, several studies have proposed that the functional link between MITF and GATA4 might affect the c-Kit^+^ cells after MI. A previous study has reported that MITF transcriptionally promotes GATA4 expression by binding to the E box in the GATA4 promoter in cardiac hypertrophy, responding to stress from ischemia [9]. MITF can also interact directly with c-Kit signaling, expressed not only in cranial neural crest derivatives and mast cells but also in cardiomyocytes [18]. For example, a previous study has clearly demonstrated that c-Kit identifies cardiac progenitors of cardiac neural crest (CNC) origin using two recombinase systems with c-Kit^CreERT2/+^ and Wnt1-Flpe recombinase driver mouse lines [18]. The c-Kit^+^ CNC-derived cardiomyocytes in c-Kit^CreERT2/+^ lineage tracing are widespread in atrial and ventricular cardiomyocytes, as well as in pericardial, endocardial, and epicardial cells [18]. Interestingly, MITF is expressed in c-Kit^+^ CNCs and their cardiomyocytic derivatives [18].

GATA4 is the zinc finger transcription factor that has demonstrated the ability to activate Bcl2 at mRNA and protein levels in adaptive responses by cardiomyocytes, helping to improve cardiomyocyte survival [19]. In another study, GATA4 overexpression in transgenic mice upregulated the level of Bcl2 in the heart and increased coronary flow reserve, as well as heightened cardiac contractility by promoting angiogenic factors such as VEGF [20]. In addition, a previous study generated a D3 mouse embryonic stem cell line with highly expressed GATA4 [21]. This cell line enhanced cardiomyocyte generation in differentiating embryoid bodies and elevated the expression of endogenous cardiac differentiation genes [21]. In another study, researchers created c-Kit^+^ cells with lentiviruses encoding four different cardiac transcription factors—GATA4, MEF3C, NKX2.5, and TBX5—alone or combined with each other [22]. No significant induction of endothelial cell markers was found in GATA4-overexpressing c-Kit^+^ cells [22].

Recently, the previous work of Dr. Piero Anversa, who studied c-Kit^+^ cardiac stem cells, has been retracted. In addition, the role of endogenous c-Kit cells still questioned whether cardiac stem cells existed. In fact, the mechanisms underlying acute MI are very different from those governing the wound repair response to MI, so the assumption that substance P should contribute to cardiac regeneration after MI is simply unfounded. Although we demonstrated that the expression of c-Kit, MITF, and GATA4 induced by SP is associated with the resident c-Kit^+^ cell expansion in LV repair post I/R, the present study is not the major focus of c-Kit, but rather SP signaling in c-Kit^+^ cells. Of note, there is difference in c-Kit, MITF, or GATA4 cellular distribution between the IA^7daysI/R^ and IA^7daysSP+I/R^ groups. The distribution of c-Kit^+^, MITF^+^, and GATA4^+^ cells in the IA^7daysI/R^ group is relatively well distributed throughout the infarct, whereas the highest concentrations of c-Kit^+^, MITF^+^, and GATA4^+^ cells in the IA^7daysSP+I/R^ group are far more focal and located within vascular structures surrounded by rings of DAPI^+^ cells. As shown in Figure 7(d), our findings did not establish a causal relationship between the SP-mediated activation of c-Kit, GATA4, and MITF and the expansion of c-Kit^+^ GATA4^high^ MITF^+^ cells in the post I/R myocardium. Nonetheless, we have clearly demonstrated by comprehensive analysis of cardiac specific markers between LV^7daysI/R^ and LV^7daysSP+I/R^ that SP, through GATA4 and MITF expression, can elevate an expansion of c-Kit^+^ GATA4^high^ cells. Overall, our results suggest that MITF might play a transcriptional regulator role linking c-Kit^+^ GATA4^high^ cells and SP-mediated c-Kit^+^ cell expansion post-MI.

## Data Availability

(1) All data used to support the findings of this study are included within the article. (2) All data used to support the findings of this study are included within the supplementary information file. (3) All data used to support the findings of this study are available from the corresponding author upon request.

## Abbreviations

CNC: Cardiac neural crest
EDCs: Explant-derived cells
HF: Heart failure
IA: Infarct-related areas
I/R: Ischemia/reperfusion injury
LV: Left ventricular
MI: Myocardial infarction
MITF: Microphthalmia-associated transcription factor
NK_1_R: Neurokinin-1 receptor
qRT-PCR: Quantitative reverse-transcription PCR
SP: Substance P.

## Supplementary Materials

Supplementary Materials Fig S1: the effects of FTY720 and RP67580 inhibitors on c-Kit cells with or without SP. (A) RT-PCR analysis of relative mRNA levels of the targeted genes in SP-stimulated c-Kit^+^ cells pretreated with one or both of these inhibitors. RPL32 is the loading control. (B) The plot densitometry graph of NK_1_R, MITF, GATA4, and c-Kit mRNA. Fig S2: FTY720 and RP67580 inhibit the expression of MITF, GATA4, and c-Kit protein in SP-treated c-Kit^+^ cells. The plot densitometry graph of NK_1_R, MITF, GATA4, and c-Kit protein that western blot analysis results for SP-stimulated c-Kit^+^ cells pretreated with one or both of these inhibitors. ^∗^*P* < 0.05 or ^∗∗^*P* < 0.01 versus corresponding controls using one-way analysis of variance (ANOVA) followed by Tukey's post hoc test.

## References

[1] French B. A., Kramer C. M. (2007). Mechanisms of post-infarct left ventricular remodeling. *Drug Discovery Today: Disease Mechanisms*.

[2] Gao X. M., White D. A., Dart A. M., Du X. J. (2012). Post-infarct cardiac rupture: recent insights on pathogenesis and therapeutic interventions. *Pharmacology & Therapeutics*.

[3] Wen Z., Mai Z., Zhang H. (2012). Local activation of cardiac stem cells for post-myocardial infarction cardiac repair. *Journal of Cellular and Molecular Medicine*.

[4] Smiljic S. (2017). The clinical significance of endocardial endothelial dysfunction. *Medicina*.

[5] Bauer A. J., Martin K. A. (2017). Coordinating regulation of gene expression in cardiovascular disease: interactions between chromatin modifiers and transcription factors. *Frontiers in Cardiovascular Medicine*.

[6] Kohli S., Ahuja S., Rani V. (2011). Transcription factors in heart: promising therapeutic targets in cardiac hypertrophy. *Current Cardiology Reviews*.

[7] Tshori S., Gilon D., Beeri R. (2006). Transcription factor MITF regulates cardiac growth and hypertrophy. *The Journal of Clinical Investigation*.

[8] Rachmin I., Amsalem E., Golomb E. (2015). FHL2 switches MITF from activator to repressor of Erbin expression during cardiac hypertrophy. *International Journal of Cardiology*.

[9] Mehta G., Kumarasamy S., Wu J. (2015). MITF interacts with the SWI/SNF subunit, BRG1, to promote GATA4 expression in cardiac hypertrophy. *Journal of Molecular and Cellular Cardiology*.

[10] Liu F., Li N., Long B. (2014). Cardiac hypertrophy is negatively regulated by miR-541. *Cell Death & Disease*.

[11] Dehlin H. M., Levick S. P. (2014). Substance P in heart failure: the good and the bad. *International Journal of Cardiology*.

[12] Hong H. S., Kim S., Lee S. (2019). Substance-P prevents cardiac ischemia-reperfusion injury by modulating stem cell mobilization and causing early suppression of injury-mediated inflammation. *Cellular Physiology and Biochemistry*.

[13] Levick S. P. (2018). Understanding the complex roles of substance P in the diseased heart. *Heart, Lung & Circulation*.

[14] Sim D. S., Kim W., Lee K. H. (2018). Cardioprotective effect of substance P in a porcine model of acute myocardial infarction. *International Journal of Cardiology*.

[15] Garandeau D., Noujarède J., Leclerc J. (2019). Targeting the sphingosine 1-phosphate axis exerts potent antitumor activity in BRAFi-resistant melanomas. *Molecular Cancer Therapeutics*.

[16] Jeong Y. M., Cheng X. W., Lee S. (2017). Preconditioning with far-infrared irradiation enhances proliferation, cell survival, and migration of rat bone marrow-derived stem cells via CXCR4-ERK pathways. *Scientific Reports*.

[17] Mauretti A., Spaans S., Bax N. A. M., Sahlgren C., Bouten C. V. C. (2017). Cardiac progenitor cells and the interplay with their microenvironment. *Stem Cells International*.

[18] Hatzistergos K. E., Takeuchi L. M., Saur D. (2015). cKit+ cardiac progenitors of neural crest origin. *Proceedings of the National Academy of Sciences of the United States of America*.

[19] Kobayashi S., Lackey T., Huang Y. (2006). Transcription factor gata4 regulates cardiac BCL2 gene expression in vitro and in vivo. *The FASEB Journal*.

[20] Bisping E., Ikeda S., Kong S. W. (2006). Gata4 is required for maintenance of postnatal cardiac function and protection from pressure overload-induced heart failure. *Proceedings of the National Academy of Sciences of the United States of America*.

[21] Laemmle L. L., Cohen J. B., Glorioso J. C. (2016). Constitutive expression of GATA4 dramatically increases the cardiogenic potential of D3 mouse embryonic stem cells. *Open Biotechnology Journal*.

[22] Al-Maqtari T., Hong K. U., Vajravelu B. N. (2017). Transcription factor-induced activation of cardiac gene expression in human c-kit+ cardiac progenitor cells. *PLoS One*.

