# Supplementary material for: Substance P Administered after Myocardial Infarction Upregulates Microphthalmia-Associated Transcription Factor, GATA4, and the Expansion of c-Kit+ Cells
Source: Stem Cells Int. 2020 Feb 10;2020:1835950. doi: 10.1155/2020/1835950 (PMC7035579; doi:10.1155/2020/1835950)
Supplement: Supplementary Materials — Fig S1: the effects of FTY720 and RP67580 inhibitors on c-Kit cells with or without SP. (A) RT-PCR analysis of relative mRNA levels of the targeted genes in SP-stimulated c-Kit+ cells pretreated with one or both of these inhibitors. RPL32 is the loading control. (B) The plot densitometry graph of NK1R, MITF, GATA4, and c-Kit mRNA. Fig S2: FTY720 and RP67580 inhibit the expression of MITF, GATA4, and c-Kit protein in SP-treated c-Kit+ cells. The plot densitometry graph of NK1R, MITF, GATA4, and c-Kit protein that western blot analysis results for SP-stimulated c-Kit+ cells pretreated with one or both of these inhibitors. ∗P < 0.05 or ∗∗P < 0.01 versus corresponding controls using one-way analysis of variance (ANOVA) followed by Tukey's post hoc test. [file 1835950.f1.docx]

**Substance P administered after myocardial infarction upregulates microphthalmia-associated transcription factor, GATA4 and the expansion of c-Kit^+^ cells**

Yun-Mi Jeong^1,3^, Xian Wu Cheng^2^, Weon Kim^1,*^

^1^Division of Cardiology, Department of Internal Medicine, Kyung Hee University Hospital, Kyung Hee University, Seoul, Republic of Korea

^2^ The Department of Cardiology, Yanbian University Hospital, Yanji, China

^3^ Department of Mechanical Engineering, Korea Polytechnic University, 237 Sangidaehak Street, Si-heung city, Republic of Korea

***Corresponding author:**

Weon Kim, MD, PhD

Division of Cardiology, Department of Internal Medicine

Kyung Hee University Hospital, Kyung Hee University

Hoegi-dong, Dongdaemun-gu, Seoul, 130-701, Republic of Korea

Tel.: +82 2 958 8176

Fax: +82 2 958 8160

E-mail address: [mylovekw@hanmail.net](mailto:mylovekw@hanmail.net)

**Materials and Methods**

**Quantitative reverse-transcription PCR (qRT-PCR)**

c-Kit^+^cells were pretreated with or without FTY720 or RP67580 or both for 2 h. Then The cells were treated with or without SP (10 nM) in the presence or absence of FTY720 (5 μM) or RP67580 (20 μg/ml) or both. The cells were incubated for 16 h at 37°C in a 5% CO_2_ incubator. cDNA was synthesized using AccuPower®RocketScript^TM^ Cycle RT PreMix (dN12) (Bioneer, DaeJeon, Korea). qRT-PCR assays were carried out with SYBR®Green Mix and the appropriate primers (Applied Biosystems), and were run on a StepOnePlus real-time PCR system (Applied Biosystems). The relative gene expression from all data was obtained using the ΔCt method with normalization versus RPL-32 as previously described [16]. The primers used were: c-Kit (Forward-AGACGTACAGATCCAGAATG, Reverse-TGCTCTTTGCTGTTACCTT); GATA4 (Forward-ACCCTGCGAGACACCCCAAT, Reverse-GTAGAGGCCACAGGCGTTGC); MITF (Forward- CATCACGCATCTTGCTACGC, Reverse- TGCATGAACTGGGCTGCCTG); BNP (Forward- GTCCTAGCCAGTCTCCAGAAC, Reverse- GAAGGCGCTGTCTTGAGACC); and RPL-32 (Forward- TGTCAAGGAGCTGGAAGTGC, Reverse-AGGCACACAAGCCATCTATTCA).

**Western blot analysis**

c-Kit^+^cells were pretreated with or without FTY720 or RP67580 or both for 2 h. Then The cells were treated with or without SP (10 nM) in the presence or absence of FTY720 (5 μM) or RP67580 (20 μg/ml) or both. The cells were incubated for 24 h at 37°C in a 5% CO_2_ incubator. The frozen samples were disrupted using the TissueLyser II (Qiagen), after which an ice-cold PRP-PREP protein extraction solution with a protease inhibitor cocktail (iNtRON Biotechnology, Inc, Seoul, Korea) was added, and the samples were homogenized with stainless steel beads (Qiagen, Cam USA). Protein concentration was assessed using a BCA-kit (Thermo Scientific, Rockford, IL, USA). An equal amount of protein (80 μg) from each sample was loaded onto 10% to 12% SDS gel, and transferred to a PVDF membrane (Merk Millipore, MA, USA). The membranes were blocked for 2 h at room temperature with 5% nonfat dry milk in PBS containing 0.1% Tween-20, and incubated with primary antibodies (1:1000 and 1:500, respectively) overnight at 4°C. After washing three times, the membranes were incubated with a horseradish peroxidase-conjugated secondary antibody (1:5000) at RT for 2 h and visualized with a chemiluminescence substrate.


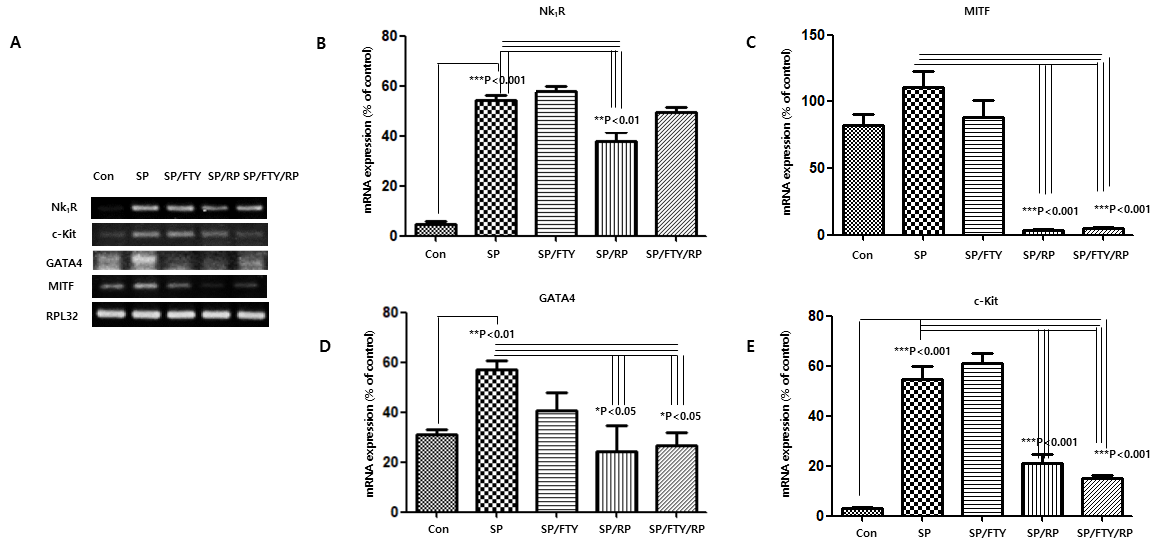


**S1. The effects of FTY720 and RP67580 inhibitors on c-Kit cells with or without SP.** (A)RT-PCR analysis of relative mRNA levels of the targeted genes in SP-stimulated c-Kit^+^ cells pretreated with one or both of these inhibitors. RPL32 is the loading control. (B)The plot densitometry graph of NK_1_R, MITF, GATA4, and c-Kit mRNA.


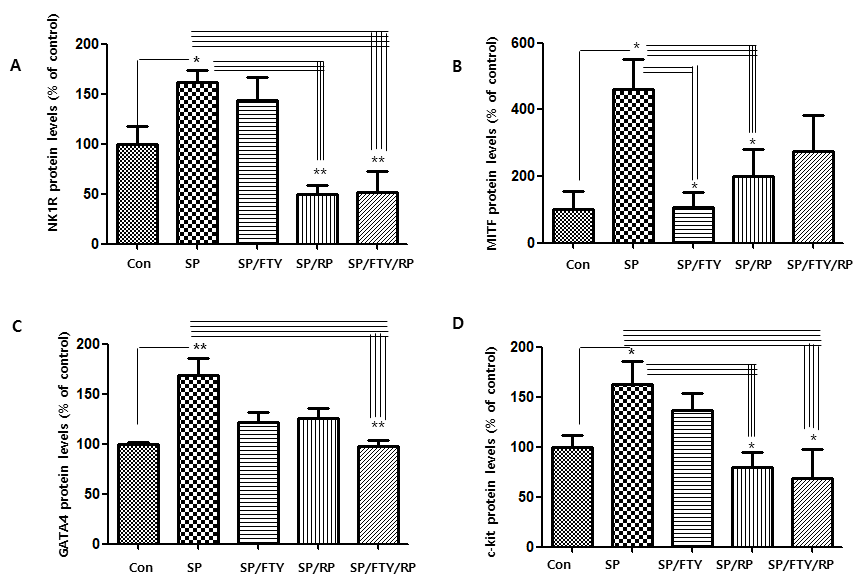


**S2. FTY720 and RP67580 inhibits the expression of MITF, GATA4, and c-Kit protein in SP-treated c-Kit cells.** The plot densitometry graph of NK_1_R, MITF, GATA4, and c-Kit protein that western blot analysis results for SP-stimulated c-Kit^+^ cells pretreated with one or both of these inhibitors. **P*<0.05 or ***P*<0.01 versus corresponding controls using one-way analysis of variance (ANOVA) followed by Tukey’s post hoc test. To determine plot densitometry graph in Fig. 6C, we used image J software.
